# Knowledge, attitude, and practice toward hypothermia in severe trauma patients among healthcare professionals in emergency departments and trauma centers in Shanghai: a cross-sectional study

**DOI:** 10.3389/fmed.2026.1629603

**Published:** 2026-01-22

**Authors:** Jun Lv, Yanyun Tan, Fei Peng, Bei Wang, Qian Liu, Zhuojuan Jiang

**Affiliations:** 1 Department of Emergency, The Second Affiliated Hospital of Naval Medical University, Shanghai, China; 2 Affiliated Huishan Hospital of Xinglin College, Nantong University, Jiangsu, China; 3 Department of Nursing, The Second Affiliated Hospital of Naval Medical University, Shanghai, China; 4 Affliated Central Hospital of Huzhou Normal University, Huzhou, Zhejiang, China; 5 Intensive Care Unit, The Second Affiliated Hospital of Naval Medical University, Shanghai, China

**Keywords:** cross-sectional study, hypothermia, knowledge, attitude and practice, medical staff, severe trauma

## Abstract

**Background:**

Hypothermia is a critical component of the trauma triad of death and has been shown to increase morbidity and mortality among severely injured patients. Early recognition and effective temperature management are essential to improving outcomes, yet clinical knowledge and implementation remain inconsistent. Understanding healthcare professionals’ preparedness for hypothermia management is therefore of great importance. To examine the knowledge, attitudes, and practices (KAP) of medical staff in emergency departments and trauma centers in Shanghai regarding the management of hypothermia in patients with severe trauma.

**Methods:**

This cross-sectional study enrolled doctors, nurses, and technicians within their emergency and trauma centers from 13 tertiary and 2 secondary hospitals in Shanghai between April and May 2023. Demographic characteristics and KAP scores were collected through a self-made questionnaire.

**Results:**

A total of 439 participants were enrolled, 192 (43.7%) were aged between 26 and 34 years, 365 (83.1%) were females, of whom 426 (97.0%) were nurses. The mean knowledge, attitude, and practice scores were 13.00 [11.00, 17.00] (possible range: 0–26), 30.00 [28.00, 34.00] (possible range: 7–35), and 28.00 [25.00, 33.00] (possible range: 9–45), respectively. Pearson correlation analysis revealed significant positive relationships between knowledge and attitude (*r* = 0.419, *p* < 0.001), knowledge and practice (*r* = 0.490, *p* < 0.001), and attitude and practice (*r* = 0.337, *p* < 0.001). Path analysis demonstrated that handling patients (*β* = −0.206, *p* < 0.001) and patient volume (*β* = 0.178, *p* = 0.001) had direct effects on knowledge. Knowledge had direct effect on attitude (*β* = 0.357, *p* < 0.001) and practice (*β* = 0.480, *p* < 0.001). Furthermore, handling patients (*β* = −0.074 and −0.097, both *p* < 0.001) and patient volume (*β* = 0.064, *p* = 0.002 and *β* = 0.085, *p* = 0.001) had indirect effects on attitude and practice, respectively.

**Conclusion:**

Medical staff in Shanghai’s emergency and trauma centers generally exhibit positive attitudes toward hypothermia management in severe trauma patients, but significant gaps remain in knowledge and practical application. These deficiencies suggest an urgent need for targeted educational interventions, incorporating evidence-based training, hands-on clinical experiences, and interdisciplinary collaboration to improve knowledge, practice, and patient outcomes.

## Introduction

Severe trauma is a leading cause of death and disability globally, predominantly due to road traffic accidents and falls from significant heights. Motor vehicle crashes alone rank eighth worldwide in mortality and tenth in disability-adjusted life years (DALYs) (1). Despite a 25% reduction in traffic accidents over the last 25 years, these incidents remain the most common cause of severe trauma (2). Annually, more than 1.3 million people die, and up to 50 million are injured on roads worldwide (3). In China, the surge in urbanization and motor vehicle usage has escalated the frequency of severe traumas resulting from traffic collisions, industrial accidents, and violent incidents. Specifically, about 203,000 traffic accidents occurred last year, with approximately 63,800 of these causing injuries or fatalities (4).

Hypothermia also plays a critical role in the outcomes of trauma patients. Known among emergency physicians and traumatologists as part of the “trauma triad of death,” which also includes coagulation disorders and acidosis, hypothermia significantly complicates trauma management (5). It adversely affects the coagulation system, constituting an independent risk factor for increased morbidity and mortality in trauma victims (6). Supporting this, a meta-analysis by Rösli et al. highlighted that accidental hypothermia at hospital admission is associated with a significantly higher mortality rate among trauma patients (7).

The Knowledge, Attitude, and Practice (KAP) model provides a theoretical framework for understanding behavior change, suggesting that knowledge is foundational, while attitude and beliefs drive the transition from knowledge to behavior (8). This theory delineates behavior modification into three distinct phases: acquiring knowledge, forming attitude or beliefs, and ultimately developing practice or behaviors (9). However, the acquisition of knowledge alone does not guarantee behavior change; it must first alter perceptions, which in turn influence behavior (10). The sequence of these steps in the KAP model is crucial, particularly in modifying the practice patterns of physicians (11). In emergency departments and trauma centers, particularly in major international cities like Shanghai, the expertise of medical staff plays a vital role in patient outcomes. The field of emergency and trauma medicine has garnered increasing focus, though gaps and inconsistencies remain in medical staff’s comprehension and management of hypothermia. Hypothermia often results from post-traumatic shock, hemorrhage, and environmental exposure, requiring a coordinated and systematic treatment approach.

However, despite the clinical importance of hypothermia in severe trauma, limited evidence is available regarding healthcare professionals’ knowledge, attitudes, and practices toward its management, particularly in high-volume urban emergency settings. Existing studies have primarily examined KAP in other medical domains and do not address hypothermia-specific competencies. To address this gap, our study provides the first assessment of KAP related to hypothermia management among emergency and trauma healthcare professionals in Shanghai. Research in various medical domains has shown that although knowledge forms the basis of competent care, positive attitudes and adequate training are essential for translating knowledge into effective clinical behaviors (8–10). Nevertheless, discrepancies between knowledge and practice have been repeatedly documented, particularly in settings where timely and accurate responses are critical, such as acute and emergency care, where guideline non-compliance remains a challenge (11). While earlier KAP investigations have primarily focused on infectious diseases or general preventive measures (8, 9, 12, 13), limited attention has been given to hypothermia management in severe trauma, despite its substantial impact on morbidity and mortality. These findings highlight the importance of assessing healthcare professionals’ KAP to identify potential barriers and inform targeted interventions for improving hypothermia management. Therefore, this study aims to evaluate the KAP toward the management of hypothermia in patients with severe trauma among healthcare professionals working in emergency departments and trauma centers in Shanghai.

## Materials and methods

### Study design and subjects

This study used a cross-sectional design and enrolled doctors, nurses, and technicians within their emergency and trauma centers from 13 tertiary and 2 secondary hospitals in Shanghai between April and May 2023. A convenience sampling method was used to recruit participating hospitals and eligible healthcare professionals. The participating institutions included 13 tertiary hospitals (Shanghai Changzheng Hospital, Shanghai Changhai Hospital, Shanghai Sixth People’s Hospital, Shanghai Jiao Tong University Ruijin Hospital, Shanghai Oriental Hepatobiliary Surgery Hospital, Shanghai Naval Characteristic Medical Center, Shanghai Tenth People’s Hospital, Shanghai First People’s Hospital, Shanghai Oriental Hospital, Xinhua Hospital Affiliated to Shanghai Jiao Tong University, Tongji Hospital Affiliated to Shanghai Tongji University, Shanghai Pudong New Area People’s Hospital, and Shanghai Huashan Hospital Affiliated to Fudan University) and 2 secondary hospitals (Tongren Hospital Affiliated to Shanghai Jiao Tong University and Jiading District Central Hospital). The inclusion criteria were: (1) medical staff working in emergency or trauma centers with clinical practice qualifications; (2) possessing knowledge relevant to trauma treatment; and (3) consenting to participate by signing an informed consent form. Participants who refused to participate in the study or provided incomplete information were excluded.

### Data collection instrument

After developing the initial questionnaire, it underwent revisions based on feedback from several experts in the relevant field. Six experts were invited to review the questionnaire, including nursing management specialists and emergency medicine professionals. They were: Zhang Weiying (Chief Nurse, Shanghai Oriental Hospital, Senior title), Hu Min (Head Nurse, Shanghai Changhai Hospital, Associate Senior), Liu Yuqing (Head Nurse, Naval Medical Center, Associate Senior), Ma Linhao (Director of Emergency Department, The Fourth People’s Hospital Affiliated to Tongji University, Associate Senior), Xu Jinghua (Head Nurse, Ruijin Hospital, Associate Senior), and Wan Jian (Director of Emergency Department/Vice President, Pudong People’s Hospital, Senior). The experts evaluated the questionnaire in terms of content relevance, clarity, clinical applicability, and coverage of hypothermia management. Revisions were made based on their suggestions until consensus was reached. A preliminary distribution involving 55 copies yielded a reliability coefficient (Cronbach’s alpha) of 0.932. The finalized questionnaire, presented in Chinese, encompasses four sections: demographic characteristics, and dimensions assessing knowledge, attitude, and practice. The knowledge dimension comprises 13 questions, scored as follows: 2 points for “very familiar,” 1 point for “heard of it,” and 0 points for “unclear,” allowing for a total score range of 0 to 26 points. The attitude dimension consists of 7 questions, utilizing a five-point Likert scale from “strongly disagree” (1 point) to “strongly agree” (5 points), with potential scores ranging from 7 to 35 points. The practice dimension includes nine questions, also based on a five-point Likert scale, from “always” (5 points) to “never” (1 point), with total scores ranging from 9 to 45 points. Adequate knowledge, positive attitude, and proactive practice are defined as scores exceeding 70% of the maximum possible score in each respective dimension (12). The questionnaire was translated into English as an Appendix. The overall Cronbach’s alpha coefficient for the questionnaire is 0.9066, with the knowledge, attitude, and practice dimensions scoring 0.9425, 0.9545, and 0.8857, respectively. The Kaiser-Meyer-Olkin (KMO) value of the total scale is 0.9167. The distribution of questionnaires to participants was facilitated using the Sojump platform. Although external validation with other established KAP instruments was not conducted, the questionnaire items were developed based on extensive literature review and expert consultation to ensure content validity and contextual relevance to hypothermia management (13).

### Statistical analysis

Data analysis was conducted using Stata 18.0 (Stata Corporation, College Station, TX, USA) and R 4.3.2. Continuous data were assessed for normality. Normally distributed data are presented as means ± standard deviations (SD), while non-normally distributed data are expressed as medians with interquartile ranges (25th–75th percentiles). Categorical data are presented as counts and percentages [*n* (%)]. For comparing two groups, the t-test was employed for normally distributed continuous variables, and the Wilcoxon Mann–Whitney test was used for non-normally distributed data. For three or more groups, ANOVA was utilized for normally distributed variables with uniform variance, while the Kruskal-Wallis test was applied to non-normally distributed data. Correlations among scores across different dimensions were analyzed using Pearson correlation coefficient for normally distributed data and Spearman correlation coefficient for data not meeting normal distribution criteria. Single-factor and multifactor regression analyses were performed to explore the relationships between demographic characteristics and scores within each dimension, selecting variables for multifactor regression based on a significance level of *p* < 0.1 in single-factor analyses. Correlations among candidate variables were examined prior to model fitting, while formal multicollinearity diagnostics and detailed checks of regression model assumptions were not systematically performed. Regression results were reported to three decimal places, with a *p*-value of less than 0.05 considered statistically significant. The structural equation modeling (SEM) was executed to assess the potential mediating effect of attitude between knowledge and practice within the KAP framework, using Stata 18.0. The SEM model’s fit was evaluated against established thresholds: Root Mean Square Error of Approximation (RMSEA) < 0.08, Standardized Root Mean Square Residual (SRMR) < 0.08, Tucker-Lewis Index (TLI) > 0.8, and Comparative Fit Index (CFI) > 0.8. If these criteria were not met, path analysis was conducted to further investigate the mediation effects. All categorical predictors included in the regression and path models were dummy coded prior to analysis.

### Ethical considerations

Ethical approval was waived by the medical ethics committee of Shanghai Changzheng Hospital, as the study involved minimal risk and the data were collected anonymously. All participants provided informed consent prior to completing the questionnaire, and all procedures adhered to the principles of the Declaration of Helsinki.

## Results

### Demographic characteristics

Among the 439 participants, 192 (43.7%) were aged 26–34 years, 365 (83.1%) were female, and 426 (97.0%) were nurses, 282 (64.2%) held junior professional and technical titles, 353 (80.4%) worked in the emergency department, and 250 (56.9%) had encountered patients with hypothermia due to severe trauma in their clinical practice. A total of 363 (82.7%) participants had managed fewer than 10 such patients. Only four participants (0.9%) were recruited from trauma centers, while the remaining participants were mainly from emergency departments, which should be considered when interpreting the representativeness of the sample. The mean knowledge, attitude, and practice scores were 13.00 [11.00, 17.00], 30.00 [28.00, 34.00], and 28.00 [25.00, 33.00], respectively. Based on the predefined cut-off value of 70% of the maximum score, 81 participants (18.5%) demonstrated adequate knowledge (≥18.2 points), 412 participants (93.8%) showed a positive attitude (≥24.5 points), and 141 participants (32.1%) reported proactive practice (≥31.5 points). Significant differences in knowledge scores were observed among participants with varying professional and technical titles (*p* = 0.007), types of hospitals (*p* = 0.014), experience with hypothermia patients due to severe trauma (*p* < 0.001), number of patient cases (*p* < 0.001), and engagement in relevant literature, guidelines, or scientific research projects (*p* = 0.005). Differences in attitude scores were most apparent across different ages (*p* = 0.004) and years of experience (*p* = 0.003). Practice scores varied significantly by gender (*p* = 0.018), occupation (*p* = 0.006), experience with hypothermia patients (*p* = 0.030), number of patient cases (*p* = 0.016), and participation in relevant scholarly activities (*p* < 0.001) (Table 1).

**Table 1:** Demographic characteristics and knowledge, attitude and practice dimensions.

Variables (*N* = 439)	*n* (%)	Knowledge	*p*	Attitude	*p*	Practice	*p*
Total score	439 (100.0)	13.00 [11.00, 17.00]		30.00 [28.00, 34.00]		28.00 [25.00, 33.00]	
Your gender			0.223		0.084		0.018
Male	74 (16.9)	13.00 [12.00, 18.00]		29.00 [28.00, 32.00]		30.00 [27.00, 34.75]	
Female	365 (83.1)	13.00 [11.00, 17.00]		30.00 [28.00, 34.00]		28.00 [24.00, 32.00]	
Your age			0.757		0.004		0.577
≤25 years old	101 (23.0)	13.00 [12.00, 16.00]		28.00 [27.00, 32.00]		29.00 [25.00, 34.00]	
26–34 years old	192 (43.7)	13.00 [11.00, 17.00]		31.00 [28.00, 34.00]		28.00 [25.00, 33.00]	
35–39 years old	70 (15.9)	13.00 [12.00, 17.00]		31.00 [28.00, 34.00]		27.00 [23.50, 32.00]	
40 years and above	76 (17.3)	13.00 [11.00, 18.00]		31.00 [28.00, 34.00]		28.50 [25.00, 33.00]	
Your work experience			0.821		0.003		0.469
≤5 years	140 (31.9)	13.00 [12.00, 16.00]		28.00 [27.00, 33.00]		29.50 [25.00, 34.00]	
More than 5 years, less than 10 years	90 (20.5)	13.00 [11.00, 18.00]		32.00 [28.00, 34.00]		28.00 [25.00, 32.00]	
More than 10 years, less than 15 years	108 (24.6)	13.00 [11.00, 16.00]		30.00 [28.00, 34.00]		27.50 [23.00, 32.00]	
≥15 years	101 (23.0)	13.00 [11.00, 18.00]		31.00 [28.00, 34.00]		28.00 [25.00, 33.00]	
Your occupation			0.436		0.450		0.006
Doctor/other	13 (3.0)	15.00 [12.00, 19.00]		32.00 [28.00, 33.00]		34.00 [31.00, 36.00]	
Nurse	426 (97.0)	13.00 [11.00, 17.00]		30.00 [28.00, 34.00]		28.00 [25.00, 33.00]	
Your professional and technical title			0.007		0.883		0.296
Primary	282 (64.2)	13.00 [11.00, 17.00]		30.00 [28.00, 34.00]		28.00 [25.00, 33.00]	
Intermediate	118 (26.9)	13.00 [11.00, 16.00]		30.00 [28.00, 34.00]		28.00 [25.00, 32.75]	
Associate Senior/Senior	13 (3.0)	19.00 [16.00, 22.00]		31.00 [28.00, 33.00]		32.00 [28.00, 36.00]	
No professional title	26 (5.9)	13.00 [9.25, 15.75]		28.50 [28.00, 33.75]		29.00 [23.00, 33.50]	
Your current department			0.712		0.720		0.488
Trauma center	4 (0.9)	16.00 [10.75, 20.25]		31.50 [28.00, 35.00]		33.50 [30.25, 35.25]	
Emergency department	353 (80.4)	13.00 [11.00, 17.00]		30.00 [28.00, 34.00]		28.00 [25.00, 33.00]	
Others (please fill in)	82 (18.7)	13.00 [11.00, 16.00]		29.00 [28.00, 34.00]		28.00 [25.00, 33.00]	
The type of hospital			0.014		0.794		0.217
Public level	13 (3.0)	13.00 [12.00, 13.00]		28.00 [26.00, 33.00]		28.00 [26.00, 31.00]	
Public secondary	121 (27.6)	13.00 [10.00, 16.00]		30.00 [28.00, 33.00]		28.00 [24.00, 34.00]	
Public third level	302 (68.8)	13.00 [12.00, 17.75]		30.00 [28.00, 34.00]		28.00 [25.00, 33.00]	
Private first-level/s-level/third-level	3 (0.7)	4.00 [2.00, 8.00]		28.00 [24.00, 31.50]		21.00 [21.00, 23.00]	
Have you ever handled patients with hypothermia caused by severe trauma in clinical work?			<0.001		0.313		0.030
Yes	250 (56.9)	13.00 [12.00, 18.00]		30.00 [28.00, 34.00]		28.00 [25.00, 34.00]	
No	189 (43.1)	13.00 [9.00, 15.00]		29.00 [28.00, 33.00]		27.00 [23.00, 32.00]	
How many patients have experienced hypothermia due to severe trauma during clinical work?			<0.001		0.063		0.016
Less than 10 cases	363 (82.7)	13.00 [10.50, 16.00]		29.00 [28.00, 33.00]		28.00 [24.00, 32.00]	
10–30 cases	56 (12.8)	16.00 [13.00, 20.00]		31.00 [28.00, 35.00]		29.50 [25.00, 34.00]	
30 or more cases	20 (4.6)	18.00 [13.75, 23.00]		31.50 [28.00, 35.00]		32.00 [27.75, 38.00]	
Are you currently participating/have participated in literature, guidelines or scientific research projects related to hypothermia caused by severe trauma?			0.005		0.427		<0.001
No	415 (94.5)	13.00 [11.00, 17.00]		30.00 [28.00, 34.00]		28.00 [25.00, 32.00]	
Yes	24 (5.5)	14.50 [13.00, 20.75]		31.00 [28.00, 34.25]		34.00 [32.00, 39.25]	

### Knowledge, attitude and practice dimensions

Analysis of the knowledge dimension revealed that the three items most frequently marked as “Unclear” were: “When the body temperature drops below 32 °C, the case fatality rate approaches 100%” (K8) at 25.1%, “Hypothermia can reduce thrombin production, inhibit fibrinogen synthesis, and impair platelet aggregation and adhesion, with these adverse effects worsening as body temperature further decreases” (K7) at 18.2%, and “Rewarming methods can range from simple, non-invasive, passive extracorporeal rewarming to active central rewarming technologies” (K10) at 17.5% (Supplementary Table S1). Attitudinal responses indicated a generally positive outlook, with over 90% of participants choosing “strongly agree” or “agree” for most statements, except for A3 and A7. For the statement, “Hypothermia is the most manageable factor in the trauma-induced ‘Triad of Death,’ and is also a factor where nursing staff can intervene directly and take the lead” (A3), 11.4% remained neutral. Regarding their confidence in effectively managing hypothermia in severe trauma cases (A7), 24.6% expressed neutral attitude, and an additional 4.8% indicated insufficient confidence (Supplementary Table S1).

In practice-related activities, 44.2% rarely attended relevant training or seminars (P7), 43.5% seldom shared updated knowledge or experiences with colleagues (P8), and 41.7% infrequently participated in relevant quality improvement projects within the hospital or community (P9). Notably, 28.9% rarely and 15.7% never used a blood warmer during transfusions involving more than 3 units of blood products (P5) (Supplementary Table S1).

### Pearson correlation analysis

Pearson correlation analysis revealed significant positive relationships between knowledge and attitude (*r* = 0.419, *p* < 0.001), knowledge and practice (*r* = 0.490, *p* < 0.001), and attitude and practice (*r* = 0.337, *p* < 0.001) (Table 2).

**Table 2:** Pearson correlation analysis.

KAP dimensions	Knowledge	Attitude	Practice
Knowledge	1.000		
Attitude	0.419 (*p* < 0.001)	1.000	
Practice	0.490 (*p* < 0.001)	0.337 (*p* < 0.001)	1.000

### Structural equation modeling and path analysis

**Figure 1:**
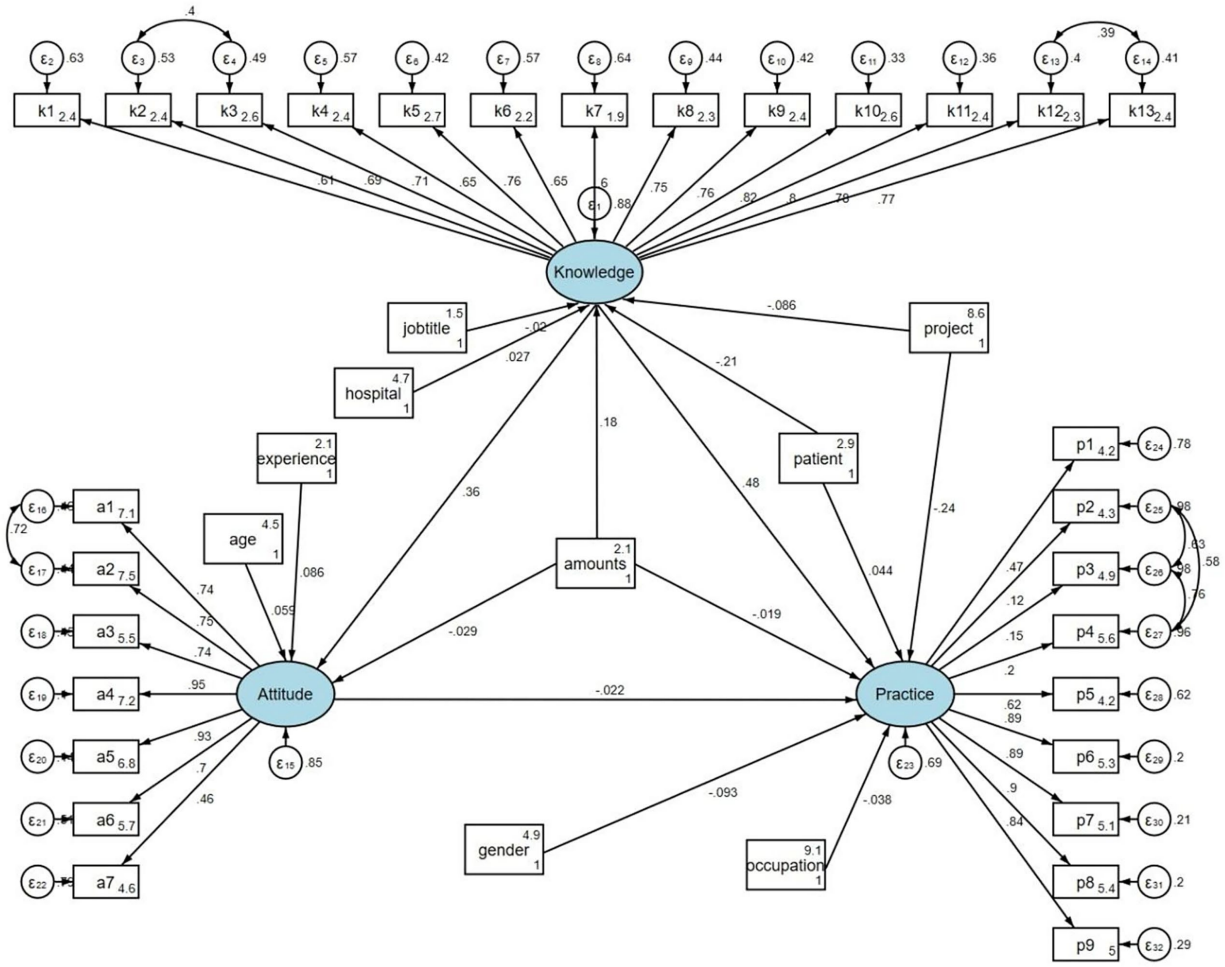
Path analysis.

The fit of the SEM indicated good model fit (RMSEA value: 0.056, SRMR value: 0.078, TLI value: 0.900, CFI value: 0.907) (Supplementary Table S2). In addition, confirmatory factor analysis was performed to examine the construct validity of the questionnaire. The CFA results indicated acceptable model fit (RMSEA = 0.066, SRMR = 0.094, TLI = 0.913, CFI = 0.921) (Supplementary Table S3; Supplementary Figure S1). Path analysis demonstrated that handling patients (*β* = −0.206, *p* < 0.001) and patient volume (*β* = 0.178, *p* = 0.001) had direct effects on knowledge. Knowledge had direct effect on attitude (*β* = 0.357, *p* < 0.001) and practice (*β* = 0.480, *p* < 0.001). Furthermore, handling patients (*β* = −0.074 and −0.097, both *p* < 0.001) and patient volume (*β* = 0.064, *p* = 0.002 and *β* = 0.085, p = 0.001) had indirect effects on attitude and practice, respectively (Table 3; Figure 1).

**Table 3:** Path analysis.

Model paths	Total effects		Direct effect		Indirect effect	
	*β* (95% CI)	*p*	*β* (95% CI)	*p*	β (95% CI)	*p*
Knowledge						
Job title	−0.020 (−0.112,0.072)	0.674	−0.020 (−0.112,0.072)	0.674		
Hospital	0.027 (−0.065,0.120)	0.562	0.027 (−0.065,0.120)	0.562		
Patient	−0.206 (−0.308,-0.104)	<0.001	−0.206 (−0.308,-0.104)	<0.001		
Amounts	0.178 (0.077,0.279)	0.001	0.178 (0.077,0.279)	0.001		
Project	−0.086 (−0.180,0.008)	0.074	−0.086 (−0.180,0.008)	0.074		
Attitude						
Knowledge	0.357 (0.247,0.468)	<0.001	0.357 (0.247,0.468)	<0.001		
Age	0.059 (−0.157,0.275)	0.591	0.059 (−0.157,0.275)	0.591		
Experience	0.086 (−0.129,0.301)	0.433	0.086 (−0.129,0.301)	0.433		
Job title	−0.007 (−0.040,0.026)	0.675			−0.007 (−0.040,0.026)	0.675
Hospital	0.010 (−0.023,0.043)	0.563			0.010 (−0.023,0.043)	0.563
Patient	−0.074 (−0.114,-0.033)	<0.001			−0.074 (−0.114,-0.033)	<0.001
Amounts	0.035 (−0.062,0.132)	0.482	−0.029 (−0.123,0.066)	0.553	0.064 (0.024,0.103)	0.002
Project	−0.031 (−0.065,0.004)	0.082			−0.031 (−0.065,0.004)	0.082
Practice						
Knowledge	0.472 (0.333,0.612)	<0.001	0.480 (0.334,0.626)	<0.001	−0.008 (−0.041,0.026)	0.646
Attitude	−0.022 (−0.115,0.071)	0.645	−0.022 (−0.115,0.071)	0.645		
Gender	−0.093 (−0.180,-0.005)	0.037	−0.093 (−0.180,-0.005)	0.037		
Age	−0.001 (−0.009,0.006)	0.726			−0.001 (−0.009,0.006)	0.726
Experience	−0.002 (−0.011,0.007)	0.691			−0.002 (−0.011,0.007)	0.691
Occupation	−0.038 (−0.124,0.047)	0.377	−0.038 (−0.124,0.047)	0.377		
Job title	−0.009 (−0.053,0.034)	0.675			−0.009 (−0.053,0.034)	0.675
Hospital	0.013 (−0.031,0.057)	0.563			0.013 (−0.031,0.057)	0.563
Patient	−0.054 (−0.154,0.046)	0.293	0.044 (−0.049,0.136)	0.354	−0.097 (−0.151,-0.044)	<0.001
Amounts	0.065 (−0.034,0.165)	0.197	−0.019 (−0.109,0.071)	0.676	0.085 (0.033,0.136)	0.001
Project	−0.284 (−0.391,-0.177)	<0.001	−0.244 (−0.340,-0.148)	<0.001	−0.040 (−0.086,0.005)	0.081

## Discussion

The study revealed that medical staff in Shanghai’s emergency and trauma centers demonstrated high agreement levels on most attitude items, indicating supportive perceptions toward hypothermia management. However, the translation of this positive attitude into consistent clinical practice remains limited, possibly due to insufficient training opportunities and a lack of standardized protocols.

The study’s findings indicate a significant positive correlation between KAP regarding hypothermia management among medical staff in emergency departments and trauma centers, supported by both correlation analyses and SEM. The SEM results affirm that greater exposure to hypothermia cases (handling patients and patient volume) directly enhances knowledge, which in turn significantly improves attitude and practice toward hypothermia management. This aligns with existing literature which suggests that direct clinical experience can enhance understanding and proficiency in specific medical interventions (14, 15). Severe trauma-induced hypothermia is part of the lethal triad, affecting coagulation and increasing mortality risk. Early management, including active rewarming and blood warmer use during transfusions, is crucial to improve outcomes (16, 17). Research also underscores the importance of multidisciplinary approaches and advanced rewarming techniques, such as heated intravenous fluids and extracorporeal warming, to effectively treat severe cases (16, 18).

Differences in knowledge and practice across professional titles, hospital types, and occupations were also observed. Given that nurses accounted for 97% of participants, potential sampling bias should be acknowledged, which may limit the generalizability of the findings. These disparities may reflect unequal access to advanced training, variations in institutional resources, and differing role expectations across clinical settings. The notable influence of professional title on knowledge suggests a correlation between advanced training and better understanding of hypothermia management, consistent with finding that highlight the impact of education levels on medical competence (19, 20). The association between professional title and knowledge might reflect differences in access to specialized training and exposure to updated clinical guidelines, potentially contributing to varying levels of knowledge among staff with different qualifications. Furthermore, the differences in practice based on gender and occupation could be related to specific role expectations or responsibilities within clinical teams, which may shape engagement in hypothermia management procedures (21, 22). Understanding how these demographic and professional factors shape both knowledge and practice can provide valuable insights for tailoring training programs to address gaps more effectively.

Age and years of experience appeared to shape staff attitudes more strongly than knowledge or practice, possibly because senior personnel tend to prioritize decision-making and coordination over hands-on procedures. This could be attributed to the rigorous standardization of training across all age groups and levels of experience, which ensures that fundamental knowledge and skills are uniformly distributed among practitioners (23, 24). Conversely, the absence of significant variations in practice based on age may be explained by the procedural consistency mandated in emergency settings. This uniformity in practice ensures that patient care quality does not fluctuate with the age or experience of the medical staff (25, 26). The study uncovered significant attitudinal differences among medical staff based on age and work experience, indicating the relationship between demographic characteristics and clinical attitudes toward hypothermia management. While procedural consistency in emergency settings ensures a stable standard of care across all staff demographics, it may also reduce opportunities for individualized decision-making and adaptation in complex cases. Additionally, the structured protocols might limit the adoption of new practices among experienced healthcare professionals. Future research could explore whether the current standardization effectively balances quality control with opportunities for clinical adaptation and growth in severe trauma care.

The analysis of the knowledge, attitude, and practice dimensions regarding hypothermia management in severe trauma patients reveals that attitudes are predominantly positive, whereas knowledge and practice show notable deficiencies. Specifically, a substantial portion of medical staff in emergency settings appears to have limited clarity on critical aspects of hypothermia, such as its profound implications on organ function and mortality, detailed mechanisms like the impact on thrombin production and platelet function, and the nuances of different rewarming techniques (27, 28). These gaps in knowledge, which notably affect both passive and active rewarming methods, are concerning given their critical role in patient outcomes. Given these insights, specific, actionable recommendations are warranted to enhance the KAP dimensions among healthcare providers. First, targeted educational programs should be developed, focusing on the less understood aspects of hypothermia management such as the physiological impacts of temperature drops and detailed rewarming strategies. For instance, simulation-based training could be particularly effective, as it allows staff to practice the application of theoretical knowledge in a controlled, realistic environment, thereby improving both knowledge and practical skills (29, 30). Additionally, it would be beneficial to integrate these topics into regular continuing medical education sessions and include them in mandatory training for all emergency department staff, ensuring that all personnel, regardless of their current level of experience or role, receive the same level of training (28, 31). Moreover, regular assessments and feedback should be incorporated to monitor knowledge retention and application in practice, helping to identify specific areas where further training may be needed (32, 33). Lastly, considering the significant role of experience in enhancing KAP scores, as indicated by statistical differences in handling patients with hypothermia, mentorship programs could be established where less experienced staff are paired with seasoned clinicians (34, 35). This approach not only facilitates hands-on learning but also helps in the transfer of tacit knowledge which is often not captured through traditional training methods (36–38).

This study has several limitations. First, the reliance on self-reported data may have introduced response bias, as participants could have overestimated their knowledge or practice behaviors. Second, although the questionnaire demonstrated high internal consistency and acceptable construct validity supported by confirmatory factor analysis, external validation with previously established KAP instruments was not conducted. This limits the comparability of our findings with those of other studies and should be addressed in future research. Third, the representativeness of the sample is limited, as most participants were nurses working in emergency departments, and only a very small proportion were recruited from trauma centers. This distribution reflects the current trauma first-aid model in Shanghai, where many hospitals do not establish independent trauma centers and trauma patients are primarily managed within emergency departments, including emergency rooms and emergency intensive care units. Fourth, the findings are specific to emergency departments and trauma centers in Shanghai, which may limit their generalizability to other regions or healthcare settings. Fifthly, part of the participants had limited clinical exposure to hypothermia cases, which may have influenced both KAP levels and the stability of associations involving patient volume. Finally, due to the cross-sectional design, causal relationships between knowledge, attitude, practice, and hypothermia management outcomes in severe trauma patients cannot be determined. Future studies should consider multi-center and longitudinal designs to enhance generalizability and to better elucidate causal pathways. In addition, intervention-based studies are warranted to evaluate the effectiveness of targeted training programs and standardized protocols in improving KAP and patient outcomes.

## Conclusion

In conclusion, although medical staff in Shanghai’s emergency departments and trauma centers exhibit generally positive attitudes, they show notable deficiencies in knowledge and practice concerning the management of hypothermia in severe trauma cases. Addressing these gaps requires a multidimensional approach, including targeted educational programs focused on theoretical and practical aspects of hypothermia management, simulation-based training to enhance clinical application, and the integration of multidisciplinary collaboration. Furthermore, mentorship programs and continuous professional development opportunities tailored to different professional roles and experience levels could enhance knowledge transfer and practical skills. Future efforts should also explore system-level interventions, such as the development of standardized protocols, the incorporation of advanced rewarming techniques, and the use of real-time feedback mechanisms to monitor and optimize clinical practices. These strategies could collectively improve the quality of care and outcomes for trauma patients experiencing hypothermia.

## Data Availability

The original contributions presented in the study are included in the article/Supplementary material, further inquiries can be directed to the corresponding author.
