# Supplementary material for: Knowledge, attitude, and practice toward hypothermia in severe trauma patients among healthcare professionals in emergency departments and trauma centers in Shanghai: a cross-sectional study
Source: Front Med (Lausanne). 2026 Jan 22;13:1629603. doi: 10.3389/fmed.2026.1629603 (PMC12872471; doi:10.3389/fmed.2026.1629603)
Supplement: Supplementary file 1 [file Supplementary_file_1.docx]

**Supplementary table 1.** Knowledge, attitude, and practice dimensions.

| **Knowledge** | **Very familiar** | **Heard about it** | **Unclear** |
| --- | --- | --- | --- |
| K1. Trauma patients whose body temperature is lower than 36 ℃ are defined as hypothermia; | 85(19.4%) | 299(68.1%) | 55(12.5%) |
| K2. Hypothermia is an independent risk factor for poor prognosis in patients with severe trauma; | 78(17.8%) | 297(67.7%) | 64(14.6%) |
| K3. Risk factors for hypothermia in emergency trauma patients include severity of trauma, prolonged exposure, large open wounds, wet clothing, hemorrhagic shock, infusion of cold liquid or blood, Endotracheal intubation, etc.; | 119(27.1%) | 279(63.6%) | 41(9.3%) |
| K4. Core temperature is the “Gold standard” For accurate body temperature measurement. Rectal or tympanic temperature measurement can be used to represent the patient’s core body temperature; | 118(26.9%) | 262(59.7%) | 59(13.4%) |
| K5. Hypothermia can cause damage to multiple organ functions, shock and even death. Maintaining hypothermia for more than 4 hours can significantly increase the mortality rate of trauma patients; | 110(25.1%) | 287(65.4%) | 42(9.6%) |
| K7. Hypothermia can lead to reduced thrombin production, inhibition of fibrinogen synthesis, and impaired platelet aggregation and adhesion, and these adverse effects will gradually worsen as the body temperature further drops; | 67(15.3%) | 292(66.5%) | 80(18.2%) |
| K8. When the body temperature drops below 32°c, the case fatality rate is close to 100%; | 69(15.7%) | 260(59.2%) | 110(25.1%) |
| K9. Hypothermia, acidosis and abnormal coagulation function are collectively called the “Triad of death” In patients with severe trauma; | 98(22.3%) | 268(61%) | 73(16.6%) |
| K10. The rewarming mode can range from simple, non-invasive, passive extracorporeal rewarming technology to active extracorporeal rewarming technology, and then to active central rewarming technology; | 57(13%) | 305(69.5%) | 77(17.5%) |
| K11. Passive rewarming techniques include reducing exposure, removing cold, wet clothing, and moving to a warmer environment; | 112(25.5%) | 275(62.6%) | 52(11.8%) |
| K12. Active external rewarming technology includes electric blankets, chemical heating pads, and air convection warming blankets; | 90(20.5%) | 281(64%) | 68(15.5%) |
| K13. Active central rewarming technology includes warm intravenous infusion, extracorporeal blood warming, and warming and humidifying oxygen; | 78(17.8%) | 283(64.5%) | 78(17.8%) |
| K14. Active rewarming has a more positive effect than passive rewarming. When the body temperature is lower than 36°c or the passive rewarming effect is not effective, active rewarming should be adopted in a timely manner; | 69(15.7%) | 300(68.3%) | 70(15.9%) |

| **Attitude** | **Strongly agree** | **Agree** | **Neutral** | **Disagree** | **Strongly disagree** |
| --- | --- | --- | --- | --- | --- |
| A1. I believe that hypothermia in patients with severe trauma is an important clinical problem requiring urgent intervention (P); | 228(51.9%) | 188(42.8%) | 21(4.8%) | 1(0.2%) | 1(0.2%) |
| A2. I believe that actively preventing hypothermia plays a vital role in the prognosis of patients with severe trauma (P); | 222(50.6%) | 197(44.9%) | 19(4.3%) | 1(0.2%) | 0 (0%) |
| A3. Hypothermia is the easiest factor to solve in the “Triad of death” Due to trauma, and it is also a factor that nursing staff can directly intervene and take the lead (P); | 185(42.1%) | 192(43.7%) | 50(11.4%) | 11(2.5%) | 1(0.2%) |
| A4. I believe that the training of medical staff in emergency departments and trauma centers on the prevention and treatment of hypothermia in severe trauma patients should be strengthened to improve their ability to respond to emergencies (P); | 210(47.8%) | 204(46.5%) | 24(5.5%) | 1(0.2%) | 0 (0%) |
| A5. I believe that improving medical staff’s ability to handle hypothermia in severe trauma patients will help reduce patient complications and mortality (P); | 211(48.1%) | 198(45.1%) | 27(6.2%) | 3(0.7%) | 0 (0%) |
| A6. I believe that medical personnel should have certain responsibilities for management and intervention of hypothermia in patients with severe trauma (P); | 177(40.3%) | 211(48.1%) | 41(9.3%) | 7(1.6%) | 3(0.7%) |
| A7. I believe that I have the ability to effectively handle hypothermia in severe trauma patients (P); | 108(24.6%) | 200(45.6%) | 108(24.6%) | 21(4.8%) | 2(0.5%) |
| **Practice** | **Always** | **Often** | **Sometimes** | **Rarely** | **Never** |
| P1. Do you regularly implement preventive measures to avoid the occurrence of hypothermia in severe trauma patients (P)? | 49(11.2%) | 120(27.3%) | 114(26%) | 127(28.9%) | 29(6.6%) |
| P2. I (we) can measure the patient’s temperature within 10 minutes of arrival (P); | 228(51.9%) | 106(24.1%) | 54(12.3%) | 45(10.3%) | 6(1.4%) |
| P3. I (we) can closely monitor changes in body temperature and keep records (P); | 232(52.8%) | 125(28.5%) | 51(11.6%) | 26(5.9%) | 5(1.1%) |
| P4. While monitoring body temperature, we (we) will closely observe various vital signs, pay attention to whether the patient has chills, and assess the patient’s subjective feeling of cold discomfort (P); | 226(51.5%) | 148(33.7%) | 43(9.8%) | 20(4.6%) | 2(0.5%) |
| P5. In any situation that requires the infusion of more than 3U of blood products, i (we) will use a blood warmer (P); | 62(14.1%) | 82(18.7%) | 99(22.6%) | 127(28.9%) | 69(15.7%) |
| P6. Do you regularly review the latest guidelines or published literature related to hypothermia in severe trauma patients (P)? | 28(6.4%) | 61(13.9%) | 125(28.5%) | 167(38%) | 58(13.2%) |
| P7. Do you regularly attend relevant training or seminars on the management of hypothermia in severe trauma patients (P)? | 25(5.7%) | 48(10.9%) | 95(21.6%) | 194(44.2%) | 77(17.5%) |
| P8. Do you share your latest knowledge or experience with colleagues regarding the management of hypothermia in severe trauma patients (P)? | 23(5.2%) | 63(14.4%) | 110(25.1%) | 191(43.5%) | 52(11.8%) |
| P9. Are you actively involved in quality improvement projects within your hospital or community regarding the management of hypothermia in severe trauma patients (P)? | 31(7.1%) | 59(13.4%) | 97(22.1%) | 183(41.7%) | 69(15.7%) |

**Supplementary table 2.** Model fit.

| **Indicators** | **Reference** | **Results** |
| --- | --- | --- |
| RMSEA | <0.080 | 0.066 |
| SRMR | <0.080 | 0.094 |
| TLI | >0.800 | 0.913 |
| CFI | >0.800 | 0.921 |

**Supplementary table 3.** Model fit indices of confirmatory factor analysis

| **Indicators** | **Reference** | **Results** |
| --- | --- | --- |
| RMSEA | <0.080 | 0.066 |
| SRMR | <0.080 | 0.094 |
| TLI | >0.800 | 0.913 |
| CFI | >0.800 | 0.921 |


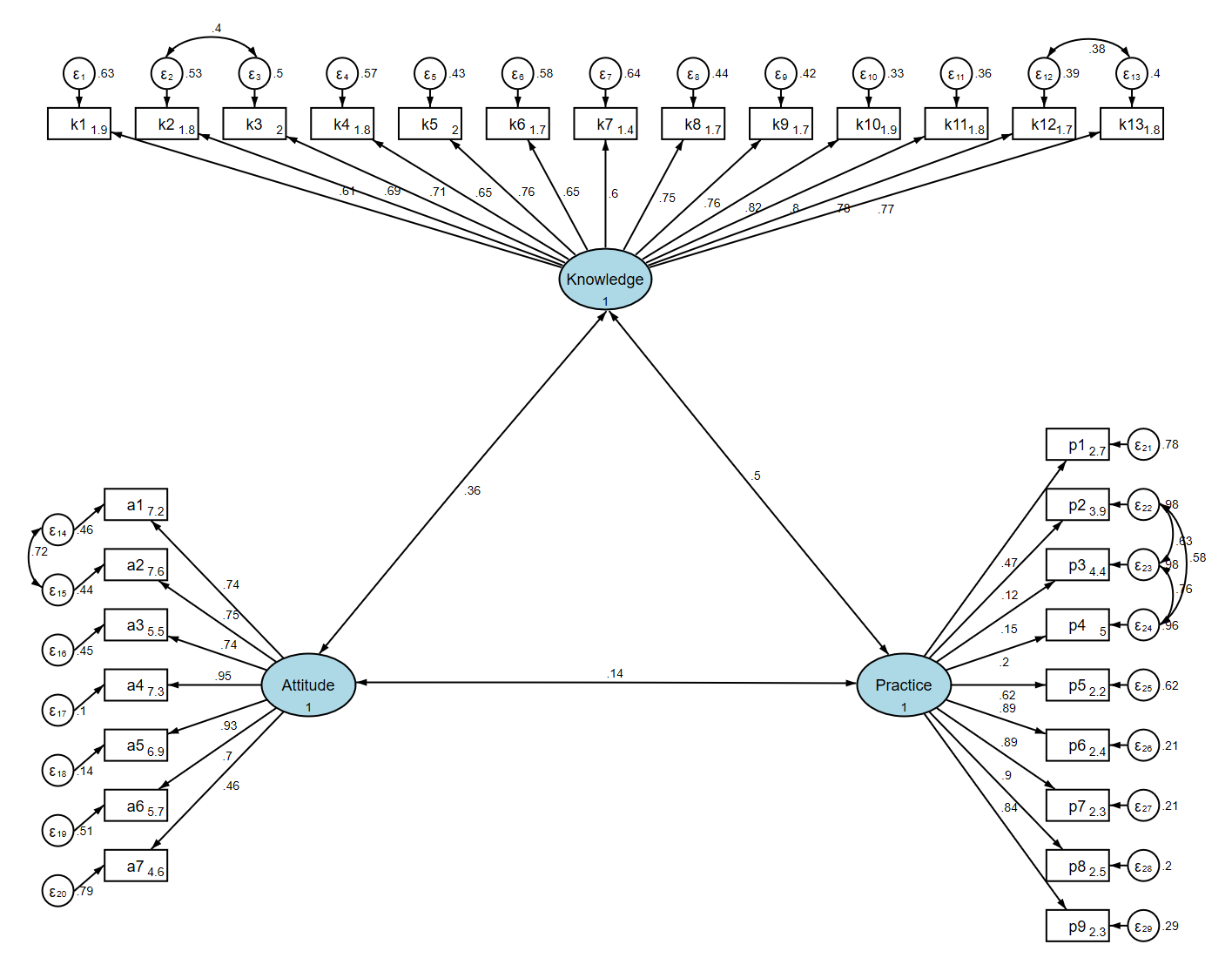


**Figure S1.** Confirmatory factor analysis model of the KAP questionnaire
